# T Helper 17 Cells in Autoimmune Liver Diseases

**DOI:** 10.1155/2013/607073

**Published:** 2013-10-10

**Authors:** Masanori Abe, Yoichi Hiasa, Morikazu Onji

**Affiliations:** Department of Gastroenterology and Metabology, Ehime University Graduate School of Medicine, Shitsukawa, To-on, Ehime 791-0295, Japan

## Abstract

Many autoimmune diseases are driven by self-reactive T helper (Th) cells. A new population of effector CD4^+^ T cells characterized by the secretion of interleukin (IL)-17, referred to as Th17 cells, has been demonstrated to be phenotypically, functionally, and developmentally distinct from Th1 and Th2 cells. Because the liver is known to be an important source of transforming growth factor-**β** and IL-6, which are cytokines that are crucial for Th17 differentiation, it is very likely that Th17 cells contribute to liver inflammation and autoimmunity. In contrast, another distinct subset of T cells, regulatory T cells (Treg), downregulate immune responses and play an important role in maintaining self-tolerance. In addition, there is a reciprocal relationship between Th17 cells and Tregs, in development and effector functions, and the balance between Th17 and Treg cells can affect the outcome of immune responses, particularly in autoimmune diseases. In this review, we will focus on the latest investigative findings related to Th17 cells in autoimmune liver disease.

## 1. Introduction

It has generally been accepted that CD4^+^ T helper (Th) cells can be categorized into two distinct subsets, that is, Th1 and Th2 cells, based on their cytokine profiles and biological functions [1]. Th1 cells are largely responsible for cellular immunity against intracellular bacteria and viruses and are distinguished by their secretion of interferon (IFN)-*γ*. Th2 cells are recognized to be integrally involved in the humoral response to parasitic infections and are defined by their characteristic secretion of cytokines of interleukin (IL)-4, IL-5, and IL-13. The pathogenic effects of Th1 cells and the protective contributions of Th2 cells have been recognized as a common feature of autoimmune diseases.

**Figure 1:**
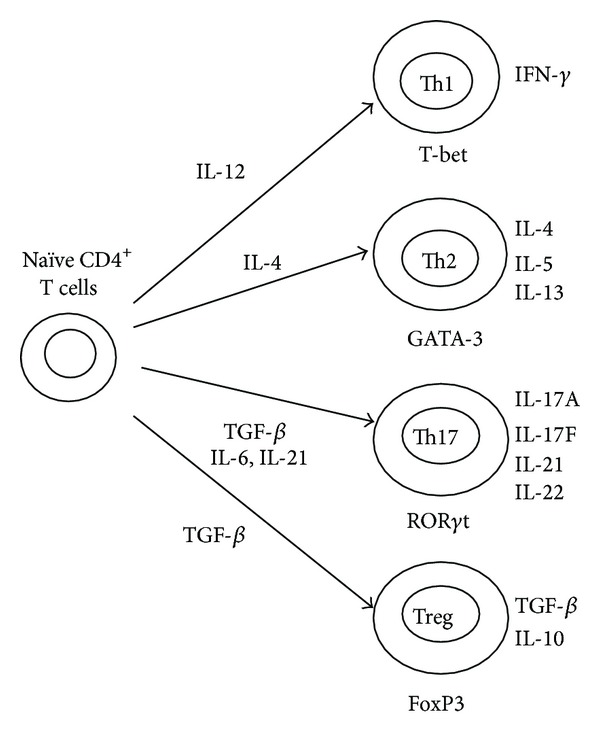
Differentiation pathways of naïve CD4^+^ T cells under different stimulation conditions. Naïve CD4^+^ T cells can differentiate into different subsets depending on the cytokine milieu. Each subset is characterized by the unique expression of transcription factors and secretion of cytokines.

Recently, a new population of effector CD4^+^ T cells characterized by the secretion of IL-17, identified as Th17 cells, has been demonstrated to be phenotypically, functionally, and developmentally distinct from Th1 and Th2 cells [2, 3] (Figure 1). In addition, another distinct subset of CD4^+^ T cells, regulatory T cells (Tregs), has been shown to downregulate immune responses through inhibition of effector cells [4]. These two subsets have been shown to have opposing effects in the immune response and may be involved in the pathogenesis of many diseases, including autoimmune diseases [5, 6]. In this review, we will focus on the latest findings related to Th17 cells in autoimmune liver disease.

## 2. Th17 Cells

Th17 cells have been implicated in host defense, inflammatory disease, tumorigenesis, autoimmune diseases, and transplant rejection, all of which are mediated by the production of several cytokines, including IL-17A, IL-17F, IL-21, and IL-22 [3, 7, 8]. IL-17A and IL-17F possess similar biological functions and bind to the same receptor complex, which is expressed by most cell types in the body. Both IL-17A and IL-17F are key cytokines in the recruitment, activation, and migration of neutrophils and monocytes and can target nonimmune cells (such as fibroblasts, endothelial cells, and epithelial cells) to induce proinflammatory mediators, including cytokines, colony stimulating factors, CC and CXC chemokines, and metalloproteinases [7–10]. IL-21 regulates the differentiation of CD4^+^ T cells into Th17 cells in an autocrine manner, thereby amplifying the Th17 responses and inducing the autocrine loop [11, 12].

Differentiation of Th17 cells requires the action of various cytokines and transcription factors. In mice, transforming growth factor (TGF)-*β* and IL-6 can induce the differentiation of naïve CD4^+^ T cells into the Th17 phenotype [13–15]. IL-21 also supports the development of Th17 cells [11, 12]. Once Th17 cells have developed, IL-23 is required for the stabilization and further expansion of these cells [13, 14, 16]. Retinoic acid-related orphan nuclear factor (ROR)-*γ*t is a transcription factor that serves as a master regulator to direct the differentiation of Th17 cells in mice [17]. The signal transducer and activator of transcription (STAT) 3 is also critical for the generation of Th17 cells [18].

Although it has been argued that human Th17 differentiation is independent of TGF-*β* signaling, subsequent studies have confirmed that, as in murine Th17 cells, TGF-*β* is indispensable for the differentiation of human Th17 cells from naïve T cells [19–21]. While TGF-*β* is essential for the induction of RORC in naïve T cells at low concentrations, the expression and function of RORC are inhibited at high concentrations of TGF-*β* [20]. Inflammatory cytokines, such as IL-6, IL-21, IL-23, and IL-1*β*, initiate human Th17 differentiation [19–21].

## 3. Relationship between Th17 and Treg Cells

Recently, Th17 and Treg cells have been shown to have opposing immunological effects, and a regulated balance between these two cell types may be crucial for the stability of immune homeostasis. Disruption of the Th17/Treg balance may lead to chronic inflammation and autoimmunity.

Treg cells produce anti-inflammatory cytokines, such as IL-10 and TGF-*β*, and suppress functional immune reactions [4]. In addition to naturally occurring, thymus-derived Treg cells, Treg cells can also be differentiated in the periphery under specific conditions. The differentiation of Treg cells may be linked to the differentiation of Th17 cells, depending on the cytokine milieu [22]. The differentiation of both Treg and Th17 cells requires TGF-*β*. The differentiation of Th17 cells requires low concentrations of TGF-*β* along with a combination of proinflammatory cytokines (such as IL-6 and IL-21), whereas high concentration of TGF-*β* in the absence of proinflammatory cytokines induce the differentiation of Th17 cells from naïve T cells [23, 24]. In addition, IL-2 and retinoic acid promote Treg cell differentiation but inhibit Th17 cell differentiation [25, 26]. These data indicate that Th17 cells and Treg cells are reciprocally regulated and can affect the outcome of immune responses, particularly in autoimmune diseases.

Forkhead box P3 (FoxP3) is a transcription factor involved in Treg cell differentiation and has characteristically high expression [27, 28]. However, under certain circumstances, FoxP3^+^ cells also express ROR*γ*t [29]. Cells coexpressing with ROR*γ*t and FoxP3 also coexpress C-C chemokine receptor 6 (CCR6), and upon activation, these cells show decreased IL-17 production relative to that of cells expressing ROR*γ*t alone, suggesting that FoxP3 antagonizes the expression and function of ROR*γ*t, thus leading to inhibition of the Th17 pathway [23, 30]. In contrast, ROR*α* inhibits FoxP3 function [31]. These findings suggest that the relationship between Th17 and Treg cells remains complex and plastic.

## 4. Th17 Cells in Autoimmune Liver Disease

Many researchers have demonstrated the importance of Th17 cells in the pathogenesis of autoimmune diseases. Specifically, the contribution of Th17 cells in experimental autoimmune encephalomyelitis, arthritis, and inflammatory bowel disease has been investigated [32–35]. In addition, high levels of IL-17 and other cytokines related to the Th17 pathway have been reported in the sera and tissues of patients with several autoimmune diseases, such as psoriasis [36] and multiple sclerosis [37].

**Figure 2:**
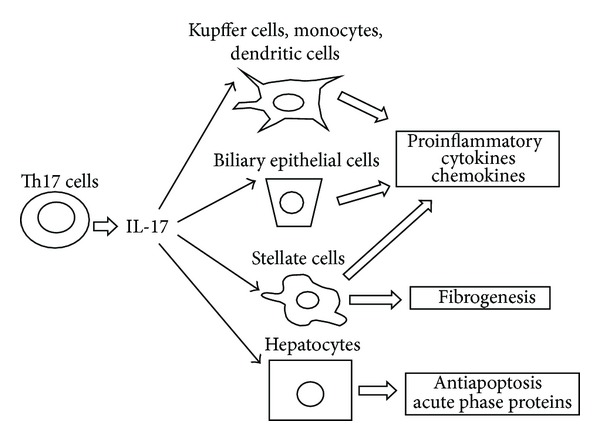
IL-17 plays a role in the pathogenesis of liver diseases. IL-17 stimulates multiple types of liver nonparenchymal cells to secrete proinflammatory cytokines and chemokines, thereby inducing and promoting liver inflammation. IL-17 also promotes liver fibrogenesis by hepatic stellate cell activation. In addition, IL-17 may stimulate hepatocytes to produce C-reactive proteins and promote hepatocyte survival.

Inflammatory responses mediated by a various immune cells play a key role in the development and progression of liver diseases. Among them, T cells are thought to be the primary effector cells contributing to the pathogenesis of many forms of liver diseases. Because the liver is known to be an important source of TGF-*β* and IL-6, Th17 differentiation may be favored in the liver. In addition, expression of the IL-17 receptor has been detected on the surface of all types of liver cells, including hepatocytes, Kupffer cells, stellate cells, biliary epithelial cells, and sinusoidal endothelial cells [38], which indicates that IL-17 may play an important role in the pathogenesis of many types of liver diseases (Figure 2). Recently, substantial evidence has been accumulated regarding the relationship between Th17 cells and liver diseases [38–41].

### 4.1. Autoimmune Hepatitis

Autoimmune hepatitis (AIH) is defined as a chronic liver disease with unknown etiological factors and is associated with aberrant autoreactivity and a genetic predisposition [42, 43]. The target antigens on the hepatocyte membrane are not known, but it is likely that liver membrane-specific activated T cells are important in the development and/or progression of the disease.

Zhao et al. [44] reported that serum IL-17 levels and the frequency of circulating Th17 cells in patients with AIH are substantially higher than those in healthy controls or patients with chronic hepatitis due to hepatitis B virus. In addition, IL-17^+^ lymphocytic infiltration (primarily of the CD4^+^ phenotype) in the liver substantially increases in AIH, and the degree of hepatic IL-17^+^ cell infiltration is positively correlated with the degree of hepatic inflammation and fibrosis in patients with AIH. IL-17 has also been demonstrated to induce IL-6 expression via the mitogen-activated protein kinase pathway in hepatocytes, thus indicating that Th17 cell proliferation is the key trigger in the pathogenesis of AIH and that the positive feedback loop between Th17 cells and hepatocytes exacerbates the inflammatory process [44].

Functional Treg cell impairment and decreased Treg cell number have been identified in patients with AIH [45–47]. Treg cell impairment in AIH varies with disease stage, appearing worse at presentation than during remission, thereby showing functional restoration potential [47]. Longhi et al. [48] reported that Treg cells can be expanded and generated *de novo* (from CD4^+^CD25^−^ cells) in patients with AIH and that the suppressor function and FoxP3 expression levels of these cells are higher than those in freshly isolated Treg cells. However, Treg cells generated from CD4^+^CD25^−^ cells in patients with AIH have been found to contain a greater population of IL-17^+^RORC^+^ cells and these cells suppressed CD25^−^ effector cell proliferation with less efficiency than Treg cells from CD4^+^CD25^high^ cells [49]. Inhibition of IL-17 or Th17 differentiation was found to lead to phenotypic and functionally stable Treg cells, suggesting that the anti-Th17 approach is an important step toward the establishment of new therapeutic strategies in AIH.

### 4.2. Primary Biliary Cirrhosis

Primary biliary cirrhosis (PBC) is a chronic cholestatic liver disease characterized by the destruction of small- and medium-sized intrahepatic bile ducts [50, 51]. Although several studies have examined the autoimmune mechanisms underlying biliary damage in PBC, the underlying cause of the disease remains largely unknown. Autoreactive CD4^+^ and CD8^+^ T cells have been implicated in the pathogenesis of PBC.

IL-2 receptor (IL-2R)^−/−^ mice spontaneously produce antimitochondrial antibodies, especially against the E2 subunit of pyruvate dehydrogenase, and develop portal inflammation with ductular damage, which is characteristic of PBC [52]. These mice have been found to have a decreased frequency of CD4^+^FoxP3^+^ Treg cells. In contrast, they showed increased serum IL-17 levels and marked aggregations of Th17 cells near the portal tracts in the liver [53].

Several studies have demonstrated a close correlation between PBC and Th17 in humans. The number of Th17 cells in peripheral blood has been found to be higher in patients with PBC than in healthy controls [54, 55]. Furthermore, IL-17 and pro-Th17 cytokines, that is, IL-1*β*, IL-6, and IL-23, were substantially upregulated in terms of both gene expression and serum concentration in patients with PBC relative to healthy controls [53, 56]. Harada et al. [56] demonstrated that liver tissues from patients with PBC have higher counts of IL-17^+^ cells per portal tract than liver tissues from normal controls, which is consistent with the results obtained for an animal model [52]. Furthermore, biliary epithelial cells possess the ability to produce pro-Th17 cytokines, such as IL-6, IL-1*β*, and IL-23, in response to pathogen-associated molecular patterns [56], suggesting that periductal IL-17-secreting cells facilitate the migration of inflammatory cells around the bile ducts. These inflammatory cells could be associated with chronic inflammation of the bile ducts in PBC. In contrast, patients with PBC possess reduced counts of Treg cells [54, 55, 57], indicating that an enhanced Th17 response and a weakened Treg response may both play an important role in the pathogenesis of PBC.

### 4.3. Primary Sclerosing Cholangitis

Primary sclerosing cholangitis (PSC) is a fibrosclerotic disease of the bile ducts, with diffuse structuring of the intrahepatic and extrahepatic biliary tree [58, 59]. The etiological factors and pathogenesis of PSC remain poorly understood, but autoimmune mechanisms are believed to contribute to the development and progression of this disease state. The biliary epithelium appears to be the target for immune-mediated injury. Recently, Katt et al. [60] reported that patients with PSC show increased numbers of Th17 cells in response to heat-inactivated pathogens, which are present in the bile duct of the majority of patients with PSC, relative to healthy controls and patients with PBC. In addition, IL-17^+^ lymphocytes were detected within the periductal areas of patients with PSC by immunohistochemical analysis. The Th17 response was induced by the selective stimulation of Toll-like receptor (TLR) 5 and TLR7 but not by stimulation of other pattern-recognition receptors.

One of the histological features of PSC is fibroobliterative sclerosis of intra- and/or extrahepatic bile ducts. Th17 cells may contribute to fibrosis thorough production of IL-17A and other cytokines. Meng et al. [61] demonstrated that the mRNA levels of IL-17A and its receptor increased in animal livers when fibrosis was induced by bile duct ligation and carbon tetrachloride and that serum IL-17A levels were associated with the development of liver fibrosis. These findings indicate that Th17 may contribute not only to inflammation but also to fibrosis in the pathogenesis of PSC. In addition, IL-17RA deletion in mice dramatically inhibits both models of liver fibrosis; therefore, IL-17 may promote liver fibrosis through hepatic stellate cell (HSC) activation or promotion of liver inflammation through the upregulation of proinflammatory cytokines and chemokines in HSC or Kupffer cells. However, the animal models used in these studies do not exhibit all of the attributes of PSC. In particular, the role played by Th17 cells in the pathogenesis of large bile ducts has not yet been clarified. Further studies using other animal models with sclerosing cholangitis and biliary fibrosis [62] are required.

### 4.4. IgG4-Related Sclerosing Cholangitis

IgG4-related sclerosing cholangitis (IgG4-SC) is a recently described biliary disease that has unknown etiological features and presents with biochemical and cholangiographic features similar to those of PSC and is often associated with autoimmune pancreatitis and other fibrotic conditions [63]. In this condition, the patient's IgG4 serum level is elevated and IgG4-positive plasma cells infiltrate into the bile ducts and liver tissue. Th2-dominant immune responses or Treg cells appear to be involved in the underlying immune reaction [64–66]. Therefore, the immunopathogenesis of IgG4-SC appears to be distinct from that of PBC and PSC. However, the role of Th17 cells in the pathogenesis of IgG4-SC has not yet been clarified, and further studies are required.

### 4.5. Th17 Cells in Liver Fibrosis

Liver fibrosis is a common outcome of chronic liver diseases, including autoimmune liver disease, and potentially leads to portal hypertension, hepatic failure, and liver cancer. Activated HSCs play a critical role in collagen and extracellular matrix production. In addition, accumulating evidence indicates that IL-17 also plays an important role in promoting liver fibrosis by inducing HSC activation [61, 67–69].

The frequency of Th17 cells in the diseased liver correlates with liver fibrosis in patients with viral hepatitis [67, 70], AIH [49], and alcoholic liver disease [71]. Furthermore, IL-17A and IL-17RA deficiency protects mice from liver fibrosis induced by CCl_4_ and bile duct ligation [61, 68, 69]. Tan et al. [68] recently reported that activation of HSC and production of collagen in CCl_4_-induced liver fibrosis are IL-17A dependent. Therefore, IL-17A neutralization may be a promising approach for antifibrotic therapy in patients with chronic liver diseases.

## 5. Conclusion

Unbalanced Th1/Th2 responses in the liver have long been proposed to be associated with perpetuated inflammation and subsequent liver fibrosis. The recently discovered Th17 cells have also been linked to host defense and autoimmunity. Although research on Th17 cells has progressed, several unanswered questions still require clarification, such as the interaction between Th17 cells and other subsets of T cells, especially Treg cells. Th17/Treg imbalance has been implicated in the pathogenesis of many diseases, especially autoimmune diseases. Elucidation of the role of Th17 differentiation and regulation will provide investigators with a novel target for the treatment of autoimmune liver disease.
